# Research on the Consumption Trend, Nutritional Value, Biological Activity Evaluation, and Sensory Properties of Mini Fruits and Vegetables

**DOI:** 10.3390/foods10122966

**Published:** 2021-12-02

**Authors:** Jiaqi Wang, Tingting Ma, Lukai Wang, Tian Lan, Yulin Fang, Xiangyu Sun

**Affiliations:** College of Enology, College of Food Science and Engineering, Northwest A&F University, Xianyang 712100, China; hello-wjq@nwafu.edu.cn (J.W.); matingting@nwafu.edu.cn (T.M.); wlk@nwafu.edu.cn (L.W.); lt771451884@nwafu.edu.cn (T.L.); fangyulin@nwsuaf.edu.cn (Y.F.)

**Keywords:** mini fruits and vegetables, consumption trend, quality, further development

## Abstract

Mini fruits and vegetables (MFV) are pocket fruits and vegetables whose shape and volume are significantly smaller than those widely sold and well-known normal fruits and vegetables (NFV) on the market. Through the research on the market status and consumption trends of MFV, it was found that MFV have recently become a new market favorite. However, compared with NFV, there was found to be no relevant data on sensory quality, nutritional value, safety, etc. of MFV; this could indicate low consumer awareness of MFV, which in turn affects their planting and sales choices, as well as the market scale remaining small. In this context, six MFV with high degree of marketization were selected and compared with their corresponding NFV to evaluate the nutritional value, biological activity, and sensory properties. The results showed the nutritional value of MFV to be mainly related to their species. The nutritional value of MFV derived from immature, tender vegetables was generally lower than that of mature NFV. For example, the content of zeaxanthin in normal maize was 0.43 mg/kg, which was about 2.87 times that of mini maize (0.15 mg/kg). For newly cultivated mini varieties, their nutritional value often had different trends and rules compared with NFV. The nutritional value obtained by consuming MFV is not equal to that obtained by consuming the corresponding NFV of the same weight.

## Highlights:

1. Nutritional and sensory qualities of mini fruits and vegetables (MFV) are reported.
2. Consumers have low awareness of MFV but hold positive attitudes toward their development.
3. MFV differ greatly from the normal variety in nutritional and sensory quality.
4. Nutritional value obtained by MFV is unequal to that of the normal variety of the same weight.
5. MFV should embrace a new route that pays equal attention to appearance, taste, and nutrition.

## 1. Introduction

Mini fruits and vegetables (MFV), as the name suggests, are pocket fruits and vegetables whose shape and volume are significantly smaller than those widely sold and well-known normal fruits and vegetables (NFV) on the market. MFV can be roughly divided into two categories: immature and newly cultivated. Immature fruits and vegetables are those that are usually picked when they are still young leaves or fruits. These MFV are often fresh and tender, but their maturity is low, and some are even unformed; therefore, their appearance is different from the mature NFV; common types of MFV include mini spinach and mini maize. The newly cultivated variety of fruits and vegetables are those that are almost the same in shape to NFV but smaller in size; common types include cherry tomatoes [1], finger carrots, and baby pumpkins [2].

In the early stage, MFV were not popular, but in recent years, with the continuous development of science and technology, a greater number of MFV have been introduced, cultivated, and planted, and the market potential is enormous. At the same time, as people’s living standards and consumption levels have been gradually improving, they have begun to pursue high-nutrition, healthy novel foods [3]. Some consumers will buy MFV because they are peculiar and lovely; other consumers prefer MFV due to the convenience of carrying and eating them rather than large NFV or due to their small family size and lower demand for fruits and vegetables.

The majority believe that MFV have similar or higher nutritional value compared with their normal counterparts. At present, the market for MFV remains small for a number of reasons. First, consumers are vague about the concept of MFV, often using size as the only evaluation criterion. Secondly, the public has many opinions on the growth and development of MFV; some people think that MFV are genetically modified foods, while others believe that all MFV are immature, tender vegetables and young fruits. Moreover, through market research, it was found that compared with NFV, the prices of MFV are generally higher, with many supermarkets even directly defining MFV as “high-grade fruits and vegetables” for sale; the prices of some mini varieties can even reach more than ten times that of normal varieties, and the high price discourages consumers [3]. In addition to cognitive errors and high prices, the unclear sensory quality and nutritional value of MFV are also vital factors restricting the development of their market.

The content of nutrients in fruits and vegetables determines their edible quality, nutritional value, and biological activity; nevertheless, the nutritional value of MFV is still controversial. On the one hand, as some nutrients are synthesized only when they are mature, several studies have shown that the nutritional value of tender MFV is low. For example, Zhao et al. [4] found that several phenolic acids, flavonoids, choline, betaine, hexose, and sucrose accumulated continuously during the development of wolfberry fruit. Qi et al. [5] observed that carotenoids increased throughout the development period of persimmon fruit. Adegbaju et al. [6] indicated that the content of alkaloids, saponins, and other substances in leafy vegetables reached the highest at the mature stage. On the other hand, some nutrients will be reduced or transformed as fruits and vegetables mature; fruits and vegetables that are in the developing stage or in the state of young buds have higher nutrient content. Sha et al. [7] found the accumulation of photosynthetic products of Fuji apples in the middle stage of development to be the largest. Drozdowska et al. [8] indicated that young shoots of red cabbage are a better source of selected nutrients and glucosinolates in comparison to the vegetable at full maturity. Ramesh et al. [9] concluded that the nutrient content of tomato was at the best level in the green mature stage.

At present, studies on cherry tomatoes and tomatoes are relatively comprehensive [1,9,10], while other varieties are basically in the blank stage. Through preliminary market research, six fruits and vegetables with high marketization degree were selected as representatives. This study systematically investigated the current consumption trends of MFV for the first time; six representative MFV were selected to evaluate their nutritional value, biological activity, and sensory properties and then compared with the corresponding NFV. The aim is to provide a theoretical basis for the production and consumption of MFV and provide consumers with scientific and correct information and knowledge.

## 2. Materials and Methods

### 2.1. Materials and Chemicals

**Figure 1:**
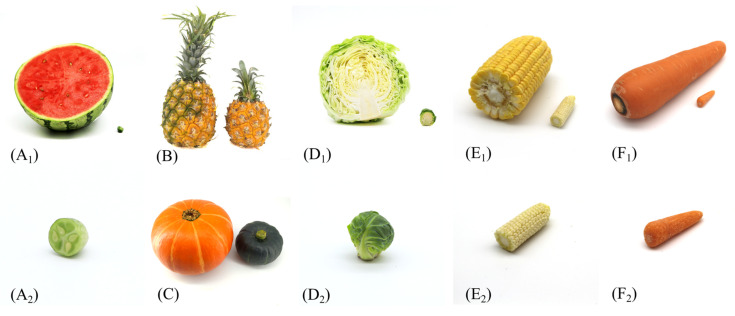
Sample photos. (**A_1_**) Normal watermelon and mini watermelon; (**A_2_**) mini watermelon; (**B**) normal pineapple and mini pineapple; (**C**) normal pumpkin and mini pumpkin; (**D_1_**) normal cabbage and mini cabbage; (**D_2_**) mini cabbage; (**E_1_**) normal maize and mini maize; (**E_2_**) mini corn; (**F_1_**) normal carrot and mini carrot; (**F_2_**) mini carrot.

Fresh, high-quality, and disease-free watermelons, pineapples, pumpkins, cabbages, maize, and carrots (mini species and normal species) were purchased from an online store (https://www.jd.com accessed on: 20 May 2020) (Figure 1). All fruits and vegetables were in the stage that could be directly processed and eaten by consumers. Gallic acid, catechin 6-hydroxy-2,5,7,8-tetramethylchroman-2-carboxylic acid (Trolox), 1,1-diphenyl-2-picrylhydrazyl (DPPH), and Folin-Ciocalteu reagent, α-amylase (bacillus subtilis source), α-glucosidase, starch soluble, 3,5-dinitrosalicylic acid (DNS), and p-nitrophenyl-α-D-glucopyranoside (PNPG) were all purchased from Shanghai yuanye Bio-Technology Co., Ltd. (Shanghai, China). All other chemicals and reagents were of analytical grade and purchased from Xilong Scientific Co., Ltd. (Guangzhou, China).

### 2.2. Questionnaire

The combination of online and offline research methods was adopted. The electronic questionnaire was designed and distributed online using survey software (https://www.wjx.cn accessed on: 20 March 2020), and the offline consumption survey was carried out nationwide. The study was conducted in accordance with the ethical guidelines of scientific research, and all participants voluntarily filled in the questionnaire under the condition of being informed. In total, 1250 questionnaires were distributed, and 1147 valid questionnaires were recovered. The questionnaire samples were randomly obtained. Further descriptive statistics was carried out on the effective questionnaire.

### 2.3. Preparation of Fruit and Vegetable Homogenate

After cutting the fructification longitudinally, the inedible parts, such as seeds and peels, were removed. Samples were mixed in different parts of the pulp and beaten by a high-speed blender (H-Ae-DNBI1, Hurom, Korea) to obtain the homogenate.

### 2.4. Physicochemical Properties

The pH values were measured using a PHS-3E pH meter (Shanghai Leici Co. Ltd., Shanghai, China). The titratable acid (TA) was determined based on the methods of Zhang et al. [11]. The total soluble solids (TSS) were determined as Bx using a PAL-1 digital Abbe Refractometer (ATAGO Co., Tokyo, Japan).

### 2.5. Nutritional and Functional Properties

The element determination was based on the method of Zhang et al. [11]. The vitamin C (Vc) content was determined using 2,6-dichloro-indophenol titration method based on the method of Lan et al. [12]. The total polyphenol content (TPC) and total anthocyanins content (TAC) were determined by Folin–Ciocalteu colorimetric method and pH differential method, and results were expressed as micrograms of gallic acid equivalents (GAE) per gram (µg GAE/g) and milligrams of cyanoside-3-glycoside (CGE) per kilogram (mg CGE/kg), respectively [10]. The method of Georgiadou et al. [13] was used to determine the β-carotene content in carrots and lycopene content in watermelon and pumpkin. The zeaxanthin content determination was based on the method by Deng et al. [14] with minor changes. After freeze-drying for 48 h, the maize grains were ground and screened for 100 mesh and sealed at −80 °C for storage. A 2.000-g sample was accurately weighed and put in a 10-mL centrifuge tube, to which 5.00 mL n-hexane-acetone solution (3:2, *v*/*v*) was added and mixed; it was then sealed at room temperature and, avoiding light, oscillated at 250 r/min for 16 h, and then, left for 4 h. A volume of 4.00 mL of supernatant was added to 1.00 mL KOH/methanol solution (20%, *w*/*v*) for 1 h under dark conditions, and then, 3.00 mL n-hexane solution and 1.00 mL Na_2_SO_4_ solution (10%, *w*/*v*) were added, mixed, and layered in a standing position under dark conditions. The absorbance of the supernatant was measured at 445 nm.

### 2.6. Biological Activities

The DPPH and ferric reducing antioxidant power (FRAP) determination were based on the method by Ma et al. [15], and the results were expressed as micromoles of Trolox equivalents per gram (μM TE/g).

The α-amylase and α-glucosidase inhibition determination were based on the method by Ismail et al. [16] with minor changes. A volume of 20 μL of phosphate buffer (PBS, 100 mM, pH 7), 10 μL of α-amylase (2 U/mL, in PBS), and 50 μL sample or acarbose (positive control, 0.1–0.5 mg/mL) were preincubated for 20 min at 37 °C in a 96-well plate. After that, 20 μL of soluble starch (1%, in PBS) was added and further incubated for 30 min at 37 °C. Then, 100 μL of the DNS color reagent was added to stop the reaction, and the mixture was boiled for 10 min. The absorbance at 540 nm was determined by a microplate reader (spark, Tecan Austria GmbH, Austria), and the results were calculated using Equation (1):

(1) $$\mathrm{Inhibition}\;(\%)=(\left(A_{test}-A_{control}\right)/A_{control})\times 100$$

where *A_test_* is the absorbance of the sample, and *A_control_* is the absorbance of PBS.

A 40-μL sample or acarbose (positive control, 0.1–0.5 mg/mL), 130 μL PBS, and 60 μL PNPG (5 mM, in PBS) were added in a 96-well plate. The initial reading (*T*_0_) was recorded at 405 nm. A 20-μL volume of α-glucosidase enzyme solution (1 U/mL, in PBS) was added to initiate the reaction, followed by incubation at 37 °C for 10 min. The final reading (*T*_10_) was recorded at 405 nm by the microplate reader, and the results were calculated using Equation (2):

(2) $$\mathrm{Inhibition}\;(\%)=\left(1-\frac{A_{test}\left(T_{10}-T_{0}\right)}{A_{control}\left(T_{10}-T_{0}\right)}\right)\times 100$$

### 2.7. Sensory Properties

The color characteristics of the samples were measured by an Ci7600 colorimeter (X-rite, Grand Rapids, MI, USA) in reflection mode. L* (lightness), a* (greenness to redness), b* (yellowness to blueness), total color difference (∆E*), and chroma (C*) were all automatically determined by the colorimeter or calculated by the respective software.

The sensory evaluation was based on the method by Ma et al. [15] with minor changes. Twenty-four professionals (12 males and 12 females, from 18 to 35 years old) from Northwest A&F University who had received sensory assessment training participated in the sensory assessment by evaluating for different attributes. After the evaluation, the average strength score of each attribute was calculated and plotted.

The E-nose assay was based on the method of Lan et al. [17] with slight modification. Five-milliliter samples were placed in 20-mL vials; testing started after equilibrating at 25 °C for 10 min, and each sample was determined at least 8 times. The parameters of the E-nose detection were as follows: the carrier gas velocity was 300 mL/min, the detection time duration was 60 s, and the cleaning time was 300 s.

The French α-ASTREE E-tongue system was used to determine the sour, sweet, bitter, salty, fresh, and full-bodied taste of the samples. The E-tongue assay was based on the method of Nakamura et al. [18] with some modification. Before using E-tongue, the sensors were activated, calibrated, and diagnosed. After 50-times dilution, the homogenization of fruits and vegetables was over 0.45-μm filter membrane sand core for suction filtration, and then, 80 mL of filtrate was taken and put into the 125-mL E-tongue burning cup in sequence. The specific parameters were as follows: sampling time 120 s, cleaning time 10 s, acquisition cycle 1 s, stirring rate 3 r/s, and parallel detection 3 times.

### 2.8. Statistical Analysis

The data obtained from the questionnaire survey were analyzed by basic descriptive statistics. The experimental results were expressed as the mean ± standard deviations (SD) of three parallel measurements. SPSS 26.0 (IBM, Armonk, NY, USA) was used to perform principal component analysis (PCA) on E-nose and E-tongue data and other data analysis. Two sample *t*-tests were calculated for pairwise comparisons. The statistical significance for all tests was set at *p* < 0.05.

## 3. Results and Discussions

### 3.1. Questionnaire Analysis

It can be seen from Appendix A that most of the respondents were 18–55 years old with generally high educational backgrounds and a diverse occupational distribution. The respondents represented 14 provinces and municipalities in China, with a large geographical span. Only a small number of respondents knew much about MFV, and even up to 40% of respondents had never heard of MFV. Among the listed MFV, the public had the greatest awareness of cherry tomatoes, accounting for 77.12%, followed by mini watermelons and mini carrots. A few respondents also knew about other MFV, such as mini ginseng fruit and *Actinidia arguta*.

The market size of MFV is generally smaller than that of NFV (Appendix A). Up to 45.45% of the respondents believed that the selling price of MFV was high, and 22.73% of those did not know the selling price; only 19.09% of the respondents thought it was very convenient to buy MFV, and only 4.58% of those often bought MFV. For common types of MFV, supermarkets and vegetable markets were the main purchase channels for people, while for rare MFV, “online shopping” was the main way to purchase them. A total of 14.38% of the respondents thought that MFV were not distinct from NFV, and there was no need to buy them. Among the purchasers, “small and lovely, rare” and “easy to eat” were the main reasons for the respondents to buy MFV. The public was generally skeptical about the safety of MFV, and their perceptions of MFV nutritional value were also vague. However, most of the respondents were interested in the research on MFV. Regarding the current situation and future development of MFV, 76.47% of respondents thought that the main factors affecting the development of MFV were insufficient popularity, followed by unreasonable price and safety issues. A total of 69.93% of the respondents supported the large-scale development of MFV, and the development prospect was relatively optimistic.

In summary, in daily life, the most common and accepted MFV are cherry tomatoes, while people’s understanding of other types of MFV is uneven. Due to the market scale, convenience, selling price, understanding, nutrition, and safety, the first choice for most consumers remains the NFV. Therefore, to promote the development of MFV, it is necessary to actively carry out research on the safety and nutrition of MFV, vigorously publicize, and change consumers’ concept. Meanwhile, it is necessary to explore ways to increase the yield of MFV, develop growers, and improve the relationship between supply and demand to further increase the market share of MFV.

### 3.2. Physicochemical Properties

**Figure 2:**
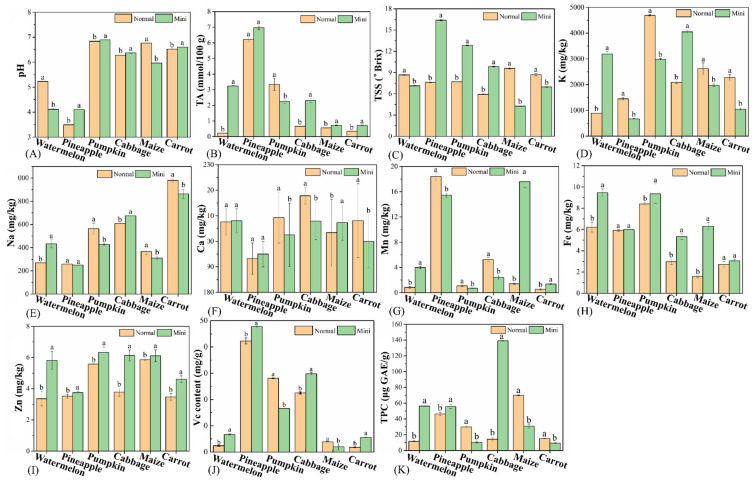
Physicochemical properties and nutrient contents of MFV and NFV. (**A**) pH; (**B**) TA; (**C**) TSS; (**D**) K; (**E**) Na; (**F**) Ca; (**G**) Mn; (**H**) Fe; (**I**) Zn; (**J**) Vc; (**K**) TPC. Values with different letters are significantly different (*p* < 0.05) from each other.

Clarify physicochemical indicators of MFV can provide basis for its processing. The pH, TA, and TSS of MFV and NFV are shown in Figure 2A,C. The pH values of MFV were significantly different from its corresponding NFV (*p* < 0.05) (Figure 2A). The pH values of mini watermelon and mini maize were significantly lower than those of normal varieties; however, the pH values of other MFV were significantly higher than those of the corresponding NFV (*p* < 0.05).

There was no significant difference between the TA of mini pineapple and normal pineapple (*p >* 0.05). TA of normal pumpkin was significantly higher than that of the mini version, while those of mini watermelon, cabbage, maize, and carrot were significantly higher than those of NFV (*p* < 0.05) (Figure 2B). Mini watermelon had 13.77 times more TA than the normal, and mini cabbage had 3.50 times more TA than the normal cabbage.

In general, TSS consists of sugar, acids, and other soluble solids, which are essential indicators of sensory quality and consumer recognition [11]. The TSS of MFV were significantly different from their corresponding NFV (*p* < 0.05), but there were no obvious rules (Figure 2C). Only the TSS of mini pineapple and mini pumpkin was greater than 10 °Brix; pineapple had the highest TSS (16.40 °Brix) among the MFV, 2.15 times that of normal pineapple. Higher TSS could make the taste of mini pineapple sweeter [19]. Maize had the highest TSS (9.60 °Brix) among the NFV, 2.25 times that of the mini maize; this might give normal maize a better taste [19].

### 3.3. Nutrient Contents

#### 3.3.1. Mineral Elements

Figure 2D,I shows the contents of six mineral elements—K, Na, Ca, Mn, Fe, and Zn—in the different cultivars of fruits and vegetables. The K and Na contents of mini watermelon and mini cabbage were significantly higher than those in NFV; the K and Na contents of several other MFV were significantly lower than those in NFV (*p* < 0.05). The K content of mini watermelon and mini cabbage were both greater than 3000 mg/kg, which were four times and two times that of normal species, respectively, making them favorable sources of K. The Ca content of different types of fruits and vegetables was between 190–220 mg/kg, and the Ca content of mini maize was significantly higher than that of normal maize (Figure 2F). Normal pineapple had the highest Mn content (18.42 mg/kg), while normal carrots had the lowest one (0.53 mg/kg) (Figure 2G). Among the same species, the Mn contents of mini corn and mini watermelon were 12.42 and 4.79 times that of their common varieties, respectively (Figure 2G).

Overall, the contents of Fe and Zn in MFV were higher than those in corresponding NFV (Figure 2H,I), indicating that mini varieties may be more superior Fe and Zn supplements. Fe and Zn deficiency in infants and young children is a common phenomenon at the age of 1–2 years [20]. On the premise of ensuring safety, compared with NFV, MFV with a small size may be good Fe and Zn supplements for infants and young children. In addition, except that the contents of six mineral elements in mini watermelon were higher than normal watermelon, there was obvious irregularity in mineral elements between other MFV and NFV.

#### 3.3.2. Vc and TPC

Vitamin C is an essential nutrient in the human diet [21]. The VC contents of different MFV and NFV are shown in Figure 2J. The results show that except for the VC contents of mini pumpkin and mini maize, which were significantly lower than those of normal varieties, the Vc contents of the MFV were significantly higher than those of the corresponding NFV (*p* < 0.05) (Figure 2J). The Vc contents of mini watermelon and mini carrots were 2.79 times and 3.00 times those of normal varieties, respectively. Pineapple had the highest Vc content in both MFV and NFV, with 47.82 mg/g and 42.30 mg/g, respectively.

Natural polyphenols are vital functional nutrients in fruits and vegetables and play an essential role in scavenging free radicals, anti-oxidation, and delaying aging [22]. There were significant differences in TPC between the six pairs of MFV and NFV (*p* < 0.05) (Figure 2K). Among the MFV, mini cabbage had the highest TPC (139.12 μg GAE/g), which was 9.76 times that of normal cabbage. This was followed by mini watermelon, for which the TPC was 56.38 μg GAE/g—4.87 times that of normal watermelon. Maize had the highest TPC (70.03 μg GAE/g) among the NFV, which was 2.27 times that of mini maize.

#### 3.3.3. Characteristic Nutrients (Carotenoids, Zeaxanthin, Lycopene, and TAC)

**Figure 3:**
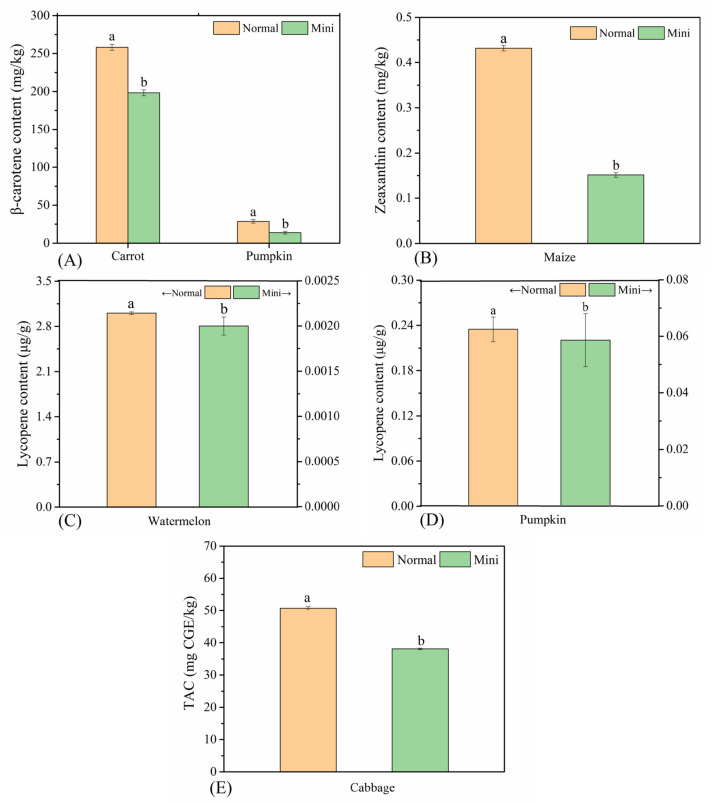
The characteristic nutrient contents of MFV and NFV: (**A**) β-carotene content of carrots and pumpkins; (**B**) zeaxanthin content of maize; (**C**) lycopene content of watermelon; (**D**) lycopene content of pumpkin; (**E**) anthocyanin content of cabbage. Values with different letters are significantly different (*p* < 0.05) from each other.

Carotenoids are a kind of effective biological antioxidants that can scavenge free radicals in the body, enhance communication between cells, and strengthen the body’s immunity. β-carotene, zeaxanthin, lycopene, etc., are common carotenoids that are consistently present in our diets and tissues [23,24]. Pumpkin and carrot are rich in β-carotenes, which are their main contributions to their orange-red or orange-yellow colors. The β-carotene contents in normal carrots and normal pumpkins were 258.15 mg/kg and 28.70 mg/kg, which was 1.30 times and 2.06 times those of mini varieties (Figure 3A). The difference was also reflected in the color of fruits and vegetables. For example, the redness of mini carrots was significantly lower than that of normal carrots, and the red was not full enough. The flesh of mini pumpkin was yellow-green, while the normal pumpkin was orange-yellow. The content of zeaxanthin in normal maize was 0.43 mg/kg, which was about 2.87 times that of mini maize (0.15 mg/kg) (Figure 3B). The research of Ndolo et al. [25] proved that zeaxanthin is mainly distributed in the endosperm of maize kernels. Since mini maize is actually an immature tender vegetable, its endosperm is incomplete or even unformed, so the content of zeaxanthin in mini maize was significantly lower. As for lycopene, normal watermelon contained 3.01 μg/g, while mini watermelon contained almost no lycopene (Figure 3C). Normal pumpkin contained 0.23 μg/g lycopene, while the level in mini pumpkin was 0.06 μg/g, which was only 26.09% that of the normal and could not be considered a good source of lycopene (Figure 3D).

Cabbage contains natural antioxidants—the anthocyanins. The results showed that the TAC of normal cabbage was significantly higher than that of mini cabbage (*p* < 0.05): 50.75 mg/kg and 38.13 mg/kg respectively (Figure 3E). This might be because mini cabbage was more tender than normal cabbage, which was still in the growth stage and immature, while anthocyanins are gradually accumulated during the whole growth stage [26], so the anthocyanin synthesis of mini cabbage was less.

### 3.4. Biological Activities

**Figure 4:**
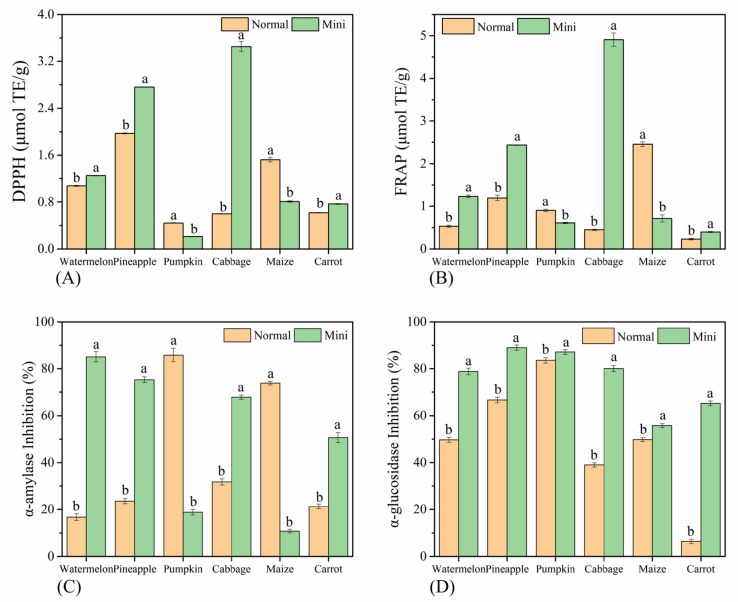
Biological activities of MFV and NFV: (**A**) DPPH radical scavenging activity; (**B**) FRAP assays; (**C**) α-amylase inhibition; (**D**) α-glucosidase inhibition. Values with different letters are significantly different (*p* < 0.05) from each other.

Fruits and vegetables are rich in nutrients, such as vitamins and polyphenols. After a specific reaction, they can remove excessive free radicals in the human body with a variety of antioxidant and other effects [27]. The antioxidant capacity of different fruits and vegetables are shown in Figure 4A,B. DPPH and FRAP of all kinds showed the same trend, and there were significant differences between mini varieties and normal varieties (*p* < 0.05). Among the MFV, the DPPH free radical scavenging rate and FRAP of cabbage, watermelon, pineapple, and carrot were significantly higher than those of the normal species (*p* < 0.05). Among them, the DPPH and FRAP values of mini cabbage were 5.76 times and 10.98 times those of normal cabbage, indicating that the antioxidant activity of mini cabbage was much higher than that of normal cabbage. Conversely, mini pumpkin and mini maize had significantly lower antioxidant levels than did the normal varieties, possibly because their normal varieties had higher levels of polyphenols and carotenoids.

Meanwhile, nutrients, such as polyphenols with antioxidant effects, are also natural inhibitors of α-amylase and α-glucosidase [28]. Due to the α-amylase and α-glucosidase that exist in the human digestive play a role in elevating blood glucose, which is a threat to diabetics [29]. Therefore, it is of great significance to inhibit the activities of the two enzymes. The inhibition rates of different fruits and vegetables on α-amylase and α-glucosidase are shown in Figure 4C,D. The results showed that the inhibition rates of MFV on the two enzymes were significantly different from those of NFV (*p* < 0.05). Sun et al. considered polyphenols to be strong inhibitors of α-amylase and α-glucosidase, consistent with our research results [30]. The TPC values of pineapple, watermelon, and cabbage have the same tendency as their inhibitory rates on α-amylase and α-glucosidase. Nevertheless, the TPC of normal carrot was higher than that of mini carrot, but its inhibition rate of the two enzymes was lower, while the TPC and α-amylase inhibition rate of normal corn and normal pumpkin was higher than that of mini varieties, but their inhibition rate of α-glucosidase was lower. This may be because the food substrates of different fruits and vegetables are quite different, which has a complex impact on the effects of biologically active substances.

These provided basic data for the varietal characteristics of MFV. In general, for MFV that were immature tender ones, such as mini cabbage, their nutritional value and biological activity were significantly lower than that of mature NFV. For the new varieties of MFV, their nutrition and biological activity were more complicated. For example, the nutritional value and biological activity of mini pineapple were significantly higher than that of ordinary varieties, the MFV of other new varieties showed no obvious regularity compared with their corresponding NFV. Overall, the nutritional value obtained by consuming NFV cannot be replaced by consuming MFV of the same weight.

### 3.5. Sensory Properties

#### 3.5.1. Color

Color, as one of the most intuitive traits of fruits and vegetables, determines their commodity value to a large extent and significantly affects consumers’ purchase intention [31]. In this study, NFV were used as controls to evaluate and analyze the color characteristics of the corresponding MFV. Table 1 provides the information about color characteristics, and there were significant differences in color between MFV and NFV (*p* < 0.05). Compared with normal varieties, a* value of mini watermelon was located in the green zone, and the difference was most significant; L* values and C* values were significantly increased, which will bring consumers a more refreshing, bright, and saturated visual experience. In addition, compared with the normal variety, the L* value of the mini pineapple was not significantly different (*p* > 0.05), but the b* and C* values were significantly higher, giving the fruit a more attractive, plump orange color. The L*, a*, b*, and C* values of mini pumpkins were significantly lower than those of normal pumpkins (*p* < 0.05), which made mini pumpkins dull in color and lacking color competitiveness. Similar to mini pumpkin, the color parameters of mini maize were significantly lower than those of ordinary maize, and the color difference (ΔE) was the largest, which showed the mini was greenish and blueish; the color was cloudy, not clear, and less saturated, and the attraction to consumers was greatly reduced. Furthermore, the total color difference (∆E) between mini carrots and normal carrots was the smallest, which was consistent with the results observed by our naked eyes.

#### 3.5.2. Artificial Sensory Evaluation

**Figure 5:**
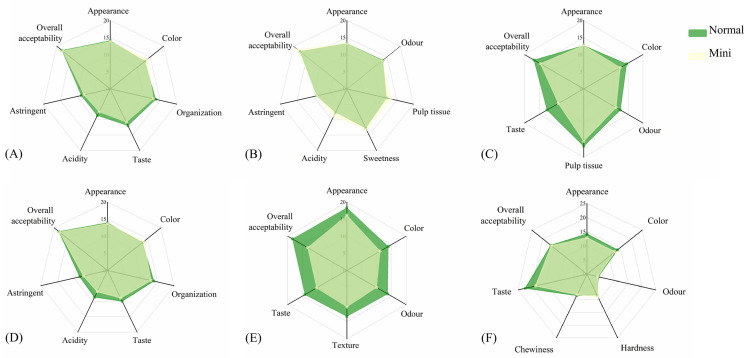
Artificial sensory evaluation of MFV and NFV: (**A**) watermelon; (**B**) pineapple; (**C**) pumpkin; (**D**) cabbage; (**E**) maize; (**F**) carrot.

Sensory properties were important reference indicators for consumers to choose fruits and vegetables, which directly affect consumers’ acceptance of food. The sensory evaluation results of 12 fruits and vegetables are shown in Figure 5. Taking into account the daily eating habits of consumers, different fruits and vegetables were treated differently. Among them, watermelon and pineapple were cut into pieces, pumpkin was steamed, and cabbage, maize, and carrots were boiled. Except for size, mini watermelon and normal watermelon had the same appearance, but the internal flesh of mini watermelon was light green, was loose and vacant, lacked the fragrance of watermelon, and had obvious acidity and astringency; the overall acceptability was significantly lower than that of normal watermelon. Compared with normal pineapple, mini pineapples had tighter flesh, higher sweetness, greater acidity, and greater overall acceptability. The skin of the mini pumpkin was dark green, the flesh color was darker, and the powdery texture was fine, but the sweetness was insufficient, the flavor and taste were inferior to normal pumpkin, and the overall acceptability was lower. The tissue state of mini cabbage was looser, the acidity and astringency of flavor were slightly higher than the normal, but there was no significant difference in overall acceptability. Mini maize kernels varied in size, with obvious gaps, and the grains were whitish, shriveled, and lackluster; the sweetness was light, and the natural fragrance of maize was inadequate. The overall acceptability was significantly lower than that of normal maize. The taste and color scores of mini carrots were lower than those of normal carrots, but they had no significant differences in their overall acceptability.

#### 3.5.3. E-Nose Analysis

E-nose is a system based on solid-state sensor; through its metal oxidation sensor, volatile compound data can be collected and outputted, and then, the food flavor profile information can quickly and non-destructively be comprehensively analyzed [32]. Figure S1(A_2_–F_2_,A_3_–F_3_) show the E-nose response of NFV and MFV, respectively; the *x*-axis was time, and the *y*-axis was relative resistivity. Each curve represented the change trend of the corresponding sensor finally reaching equilibrium with time. All samples reached equilibrium within 50–60 s. The sensitivities and response values of NFV and MFV to different sensors were different. In general, except for mini watermelon and normal maize, which showed the strongest response signal at S2 (broad-range sensitivity, very sensitive to nitrogen oxides), the other samples all showed the strongest response at S7 (sensitive to many sulfuric organic compounds and terpenes). In addition, some samples, such as normal pineapple, normal maize, etc., also had high responses to S6 (sensitive to methane, broad range) and S8 (sensitive to alcohol, aromatic compounds with broad range), while the response values of other sensors hardly changed. Figure S1A_1_–F_1_ are the radar chart of E-nose response data of samples. It was found that there was a big difference in the response value of different sensors between the normal varieties of watermelon, cabbage, maize, and carrot and the mini varieties, while normal and mini varieties of pineapple and pumpkin were closer.

**Figure 6:**
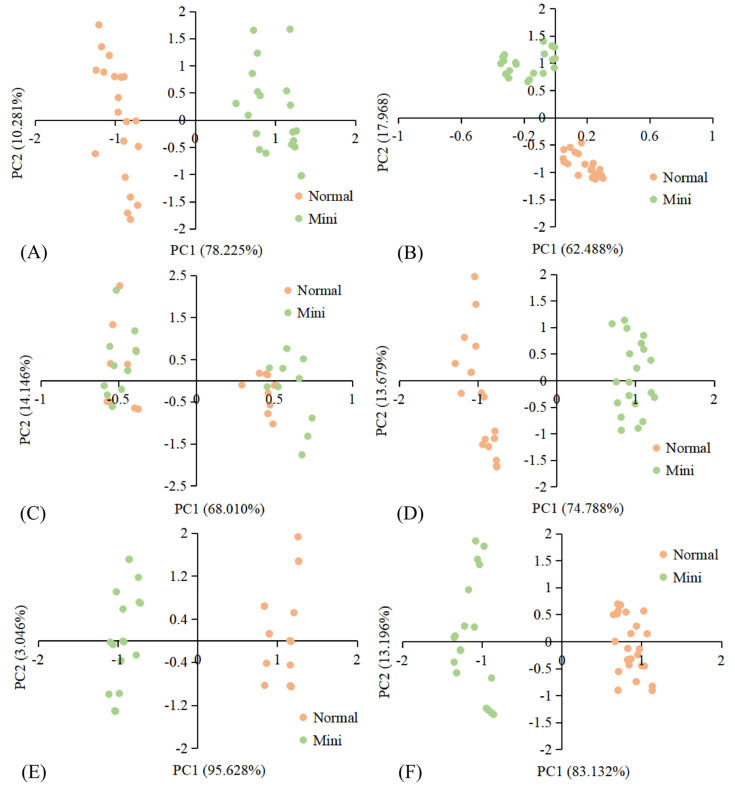
E-nose PCA score charts of MFV and NFV: (**A**) watermelon; (**B**) pineapple; (**C**) pumpkin; (**D**) cabbage; (**E**) maize; (**F**) carrot.

Principal component analysis (PCA), which uses the idea of dimension reduction to remove overlapping parts of numerous information and transform multiple variables into a few unrelated comprehensive variables, has been widely used in the comprehensive evaluation of food quality [17]. The PCA results of different fruits and vegetables based on E-nose response data were shown in Figure 6A–F_;_ the cumulative variance of the first two principal components was over 80.456%, which could represent the overall information of the samples. The results declared that, except for pumpkins (Figure 6C), there was no cross area between the other varieties of MFV and NFV; the distinction was clear, and this evidenced by finding no obvious differences in the overall flavor characteristics between these MFV and the corresponding NFV, which could be well distinguished by E-nose. In contrast, the distribution of mini pumpkin and normal pumpkins overlapped, indicating that their aroma characteristics were close to each other. The above results were consistent with the conclusions of the corresponding radar chart (Figure S1A_1_–F_1_).

#### 3.5.4. E-Tongue Analysis

E-tongue is a taste integrated sensor based on multi-sensor array (Table S1) and multivariate statistical analysis method to detect liquid samples. It is mainly used in process monitoring, freshness evaluation and shelf-life investigation, food identification, adulteration identification, and other quality control research [33].

**Figure 7:**
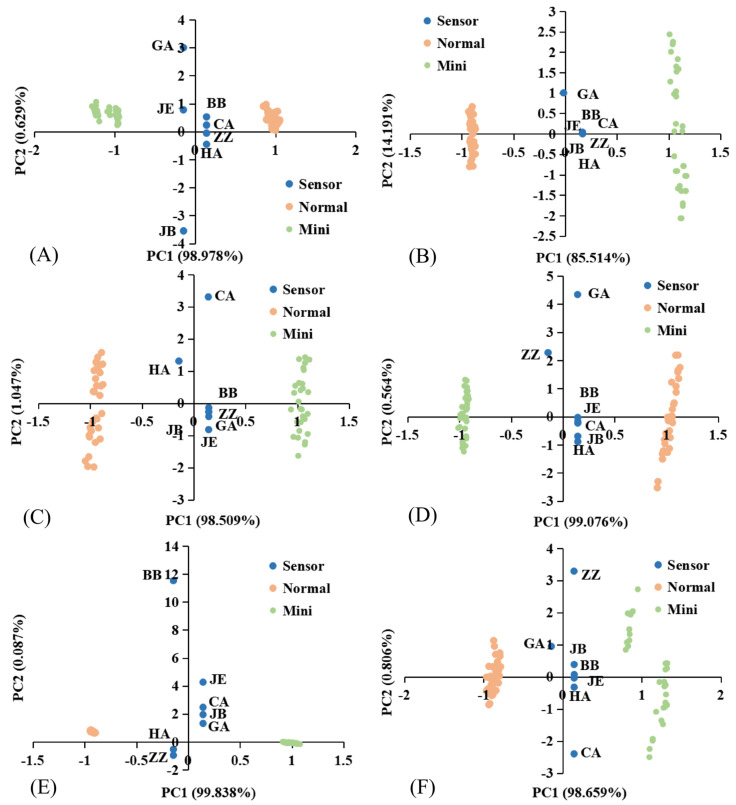
E-tongue PCA score charts of MFV and NFV: (**A**) watermelon; (**B**) pineapple; (**C**) pumpkin; (**D**) cabbage; (**E**) maize; (**F**) carrot.

Figure S2(A_2_–F_2_,A_3_–F_3_) show the E-tongue response of fruits and vegetables. All samples reached equilibrium within 100–120 s and the response signal values of the sensor CA were the largest, indicating the sourness and sweetness were the most obvious tastes of all samples. The E-tongue response data of fruits and vegetables were further analyzed by PCA (Figure 7A–F). The cumulative variance of the first two principal components of all samples exceeded 99.465%, which could represent the overall information of the samples. The results showed that there was no cross region between the samples of MFV and NFV, which indicated that there were significant differences in sour, sweet, salty, bitter, and fresh tastes between MFV and the corresponding NFV, which could be well distinguished by E-tongue. In addition, the scores of normal watermelon and mini watermelon in PC2 were both positive values, while the scores of watermelons in PC1 were different. Therefore, the loads of PC1 and PC2 of sensors GA, JE, BB, and CA could distinguish mini watermelon from normal watermelon. Among them, GA and JE contributed more to the mini watermelon, while BB and CA contributed more to the normal watermelon. Compared with sensors GA and JE, BB and CA were sensors that were more sensitive to sweetness. Therefore, the sweetness of mini watermelon was weaker than that of normal watermelon. Similarly, it can be found that the sourness of mini pineapple, normal cabbage, and normal carrot was more prominent. The results were consistent with the radar chart below (Figure S2A_1_–F_1_).

Sensory properties showed the difference of taste between MFV and NFV and provided scientific reference for consumers.

## 4. Conclusions

Through questionnaire survey and analysis, it was found that there is still a significant gap between the market size of MFV and NFV, and the popularity of MFV needs to be improved. Nevertheless, most of the respondents support the large scale of MFV and hold a positive attitude towards its development. In addition, the physicochemical, nutritional, sensory characteristics, and biological activity of six different types of MFV and NFV were compared and analyzed. The results showed that only the mini pineapple had characteristics that were nearly all better than those of the common species. Mini cabbage, mini maize, etc., had fewer nutrients and lower biological activity than their NFV counterparts. Mini watermelon, mini pumpkin, etc., which looked like NFV but belonged to the new species, were found to have no obvious differences in terms of their nutrients and biological activity compared with NFV. In terms of the sensory characteristic, the aroma and taste characteristics of MFV were often significantly different from those of NFV, but the overall acceptability of other mini varieties was inferior to that of the normal species, with the exception of mini pineapple.

Presently, people’s pursuit of food is becoming more and more diversified, and various novel products are also endlessly emerging. However, the cases in this study show that the current psychology of curiosity is the main driving force for the consumption of MFV. Most consumers are not aware of the details of MFV, such as their sources, nutritional value, and taste; MFV are not as nutritious and tasty as expected. The psychological expectation of most consumers is that MFV have the same or much higher nutritional value compared with NFV. In fact, among the six highly active MFV in the market, only mini pineapple has better nutritional value, biological activity, and sensory performance than normal pineapple. The great majority of the species are far from meeting consumer requirements. Thus, should the future development direction of MFV continue to follow the current route and attract consumers’ attention through marketing tactics, or focus on improving the nutrition and organoleptic quality base on the superior appearance? The latter one is bound to be a long-term and difficult process, but this research still firmly believes that the new route of “beautiful plus nutritious plus delicious” should be taken. Attention should be paid to the related introduction, breeding, and cultivation, especially to abandon the current method of using other plants that are similar in appearance but are actually different species. At the same time, expansion of the market scale, standardization of the market management, and provision of more information about the mini products is required. The MFV industry has the most potential to thrive with these strategies Otherwise, consumers are left to buy them at a high price due to their curiosity and then may refuse to re-purchase once the corresponding taste and nutritional value is learned and does not meet their psychological expectations. Under the development direction of “beautiful plus nutritious plus delicious”, when all MFV can be as successful as the most popular MFV—cherry tomato—the MFV industry will truly flourish. In summary, the study provided basic data and theoretical guidance for the popularization, planting, and sales of MFV, which can help promote the further development of the MFV industry.

## Figures and Tables

**Table 1:** Color parameters.

Samples		Color				
		L*	a*	b*	∆E	C*
Watermelon	Normal	29.69 ± 0.97 ^b^	13.47 ± 1.06 ^a^	6.15 ± 0.93 ^b^	0.00 ± 0.00 ^b^	14.81 ± 1.06 ^b^
	Mini	55.66 ± 0.10 ^a^	−7.03 ± 0.57 ^b^	26.49 ± 0.88 ^a^	38.84 ± 0.33 ^a^	27.41 ± 0.81 ^a^
Pineapple	Normal	42.71 ± 0.86 ^a^	−3.75 ± 0.26 ^b^	9.12 ± 0.53 ^b^	0.00 ± 0.00 ^b^	9.86 ± 0.48 ^b^
	Mini	42.05 ± 1.02 ^a^	−2.38 ± 0.05 ^a^	17.22 ± 1.79 ^a^	8.24 ± 0.36 ^a^	17.38 ± 0.46 ^a^
Pumpkin	Normal	46.89 ± 0.58 ^a^	21.30 ± 0.58 ^a^	37.40 ± 0.60 ^a^	0.00 ± 0.00 ^b^	43.04 ± 0.55 ^a^
	Mini	44.23 ± 0.39 ^b^	11.68 ± 0.36 ^b^	31.61 ± 0.55 ^b^	11.54 ± 1.60 ^a^	33.70 ± 0.51 ^b^
Cabbage	Normal	42.17 ± 0.35 ^b^	−5.73 ± 0.97 ^a^	13.61 ± 0.59 ^b^	0.00 ± 0.00 ^b^	14.78 ± 0.72 ^b^
	Mini	54.24 ± 0.16 ^a^	−6.42 ± 0.01 ^b^	22.71 ± 0.00 ^a^	15.13 ± 1.49 ^a^	23.60 ± 0.02 ^a^
Maize	Normal	77.92 ± 0.37 ^a^	5.34 ± 0.99 ^a^	42.83 ± 3.39 ^a^	0.00 ± 0.00 ^b^	43.16 ± 2.84 ^a^
	Mini	47.65 ± 0.37 ^b^	−2.85 ± 0.32 ^b^	10.28 ± 2.09 ^b^	45.20 ± 1.15 ^a^	10.68 ± 1.61 ^b^
Carrot	Normal	47.22 ± 0.55 ^a^	24.03 ± 1.10 ^a^	28.72 ± 1.32 ^a^	0.00 ± 0.00 ^b^	37.45 ± 1.39 ^a^
	Mini	46.70 ± 0.58 ^a^	21.07 ± 0.74 ^b^	28.02 ± 1.02 ^a^	3.08 ± 0.22 ^a^	35.06 ± 1.03 ^b^

^a^ The datas were expressed as the mean ± (SD) of three parallel measurements. ^b^ Values with different letters are significantly different (*p* < 0.05) from each other.

## Data Availability

The datasets generated for this study are available on request to the corresponding author.

## Supplementary Materials

The following are available online at https://www.mdpi.com/article/10.3390/foods10122966/s1, Figure S1: E-nose assay of MFV and NFV. (A_1_–F_1_) Radar charts of E-nose response data; (A_2_–F_2_) Sensor responses recorded using ten sensors of the E-nose for NFV; (A_3_–F_3_) Sensor responses recorded using ten sensors of the E-nose for MFV. (A. Watermelon; B. Pineapple; C. Pumpkin; D. Cabbage; E. Maize; F. Carrot.). Figure S2: E-tongue assay of MFV and NFV. (A_1_–F_1_) Radar charts of E-tongue response data; (A_2_–F_2_) Sensor responses recorded using ten sensors of the E-tongue for NFV; (A_3_–F_3_) Sensor responses recorded using ten sensors of the E-tongue for MFV. (A. Watermelon; B. Pineapple; C. Pumpkin; D. Cabbage; E. Maize; F. Carrot.). Table S1: Detection thresholds of each sensor of electronic tongue.

## Appendix A. Questionnaire

1. What is your age?	
A. ≤18	5.26%
B. 19–25	53.02%
C. 26–35	15.93%
D. 36–45	17.29%
E. 46–55	6.88%
F. >55	1.62%
2. What is your occupation?	
A. Student	69.64%
B. Office worker	21.86%
C. Individual business	2.43%
D. Farmer	1.62%
E. Retiree	0.40%
F. Other	4.05%
3. Where do you live and work? (Fill in the blank, please.)	

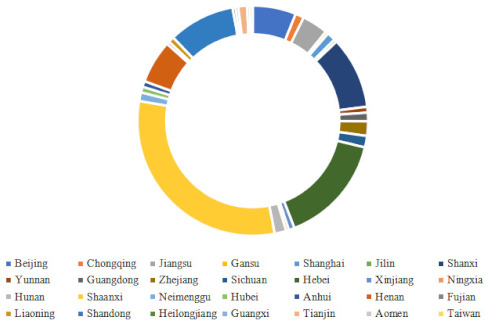

4. What is your educational background?	
A. Short-cycle Courses and Under	16.19%
B. Undergraduate	70.04%
C. Master Degree Candidate	9.31%
D. Doctoral Candidate	4.45%
E. Other	0.00%
5. How much do you know about mini fruits and vegetables?	
A. Know a lot	3.64%
B. General acquaintance	58.30%
C. Never heard	38.06%
6. What is the market size of mini fruits and vegetables in your area?	
A. Large scale, basically equivalent to normal fruits and vegetables.	4.58%
B. Medium scale, but still significantly smaller than normal fruits and vegetables.	52.29%
C. Almost no market.	43.14%
7. What mini fruits and vegetables do you know? (Multiple choice)	
A. Mini watermelon	52.29%
B. Mini carrot	37.91%
C. Baby pumpkins	30.07%
D. Mini maize	22.88%
E. Mini pineapple	20.26%
F. Miniature cabbage	12.42%
G. Cherry tomato	77.12%
H. Other	2.61%
8. Do you think mini fruits and vegetables are safe?	
A. Very safe	18.95%
B. Not sure	79.74%
C. Unsafe	1.31%
9. Do you have any experience in buying mini fruits and vegetables?	
A. Buy often	4.58%
B. Buy occasionally	67.32%
C. Never	28.10%
10. How do you buy mini fruits and vegetables? (Multiple choice)	
A. Supermarket	88.18%
B. Vegetable market	41.82%
C. Roadside vendors	15.45%
D. Buy online	22.73%
E. Other	0.91%
11. Do you find it convenient to buy mini fruits and vegetables?	
A. Very convenient	19.09%
B. Okay	68.18%
C. Not convenient	12.73%
12. Why do you usually choose to buy mini fruits and vegetables?	
A. Nutritious	18.18%
B. Convenient	50.91%
C. Small, lovely, and rare	70%
D. Other	5.45%
13. What aspects do you value when buying mini fruits and vegetables? (Multiple choice)	
A. Freshness	15.24%
B. Nutritional value	18.18%
C. Reasonable price	50.91%
D. Clean and sanitary	70.00%
E. Easy to purchase	5.45%
14. Do you think the nutritional value of mini fruits and vegetables is different from normal fruits and vegetables?	
A. Mini fruits and vegetables have higher nutritional value.	5.88%
B. Normal fruits and vegetables have higher nutritional value.	5.23%
C. The two are almost the same.	46.41%
D. Don’t know much about it.	42.48%
15. What do you think of the selling price of mini fruits and vegetables?	
A. High	45.45%
B. Normal	30.91%
C. Underpriced	0.91%
D. Unclear	22.73%
16. What is your attitude about the research on nutrition of mini fruits and vegetables?	
A. Pay close attention.	11.11%
B. General attention.	46.41%
C. Don’t focus on.	42.48%
17. What is your opinion on mini fruits and vegetables?	
A. There is no difference except for the size between mini fruits and vegetables and normal fruits and vegetables, so there is no need to buy.	14.38%
B. Mini fruits and vegetables can replace the normal with high nutritional value.	1.96%
C. Convenient to eat and suitable for a small families to eat.	32.68%
D. Small and lovely, loved by children.	47.71%
E. Other.	3.27%
18. What factors do you think hinder the development of mini fruits and vegetables?	
A. Expensive	40.52%
B. Not easy to buy	27.45%
C. Unsafe	38.56%
D. Insufficient popularity	76.47%
E. Other	3.27%
19. Do you support the large-scale development of mini fruits and vegetables in the market?	
A. Positive	69.93%
B. Negative	30.07%

## References

[1] Rapa M., Ciano S., Ruggieri R., Vinci G. (2021). Bioactive compounds in cherry tomatoes (*Solanum lycopersicum* var. *Cerasiforme*): Cultivation techniques classification by multivariate analysis. Food Chem..

[2] Sánchez E.S., Butzler T.M., Stivers L.J., Elkner T.E., Bogash S.M., Oesterling R.E., Orzolek M.D. (2012). Pennsylvania Statewide Winter Squash Cultivar Evaluation. HortTechnology.

[3] Marsola C.M., Carvalho-Ferreira J.P., Cunha L.M., Jaime P.C., Cunha D.T. (2021). Perceptions of risk and benefit of different foods consumed in Brazil and the optimism about chronic diseases. Food Res. Int..

[4] Zhao W., Zhang Y., Shi Y. (2021). Visualizing the spatial distribution of endogenous molecules in wolfberry fruit at different development stages by matrix-assisted laser desorption/ionization mass spectrometry imaging. Talanta.

[5] Qi Y., Wu H., Liu J., Chen L., Jiang Z., Zhang Y., Tian X., Li R., Yang Y., Ren X. (2021). Lycopene β-cyclase plays a critical role in carotenoid biosynthesis during persimmon fruit development and postharvest ripening. Sci. Hortic..

[6] Adegbaju O.D., Otunola G.A., Afolayan A.J. (2019). Proximate, mineral, vitamin and anti-nutrient content of Celosia argentea at three stages of maturity. S. Afr. J. Bot..

[7] Sha J., Wang F., Xu X., Chen Q., Zhu Z., Jiang Y., Ge S. (2020). Studies on the translocation characteristics of 13C-photoassimilates to fruit during the fruit development stage in ‘Fuji’ apple. Plant Physiol. Biochem..

[8] Drozdowska M., Leszczyńska T., Koronowicz A. (2020). Young shoots of red cabbage are a better source of selected nutrients and glucosinolates in comparison to the vegetable at full maturity. Eur. Food Res. Technol..

[9] Ramesh K.V., Paul V., Pandey R. (2021). Dynamics of mineral nutrients in tomato (*Solanum lycopersicum* L.) fruits during ripening: Part I—On the plant. Plant Physiol. Rep..

[10] Mustapha A.T., Zhou C. (2021). Novel assisted/unassisted ultrasound treatment: Effect on respiration rate, ethylene production, enzymes activity, volatile composition, and odor of cherry tomato. LWT.

[11] Zhang H., Zhao Q., Lan T., Geng T., Gao C., Yuan Q., Zhang Q., Xu P., Sun X., Liu X. (2020). Comparative Analysis of Physicochemical Characteristics, Nutritional and Functional Components and Antioxidant Capacity of Fifteen Kiwifruit (*Actinidia*) Cultivars—Comparative Analysis of Fifteen Kiwifruit (*Actinidia*) Cultivars. Foods.

[12] Lan T., Bao S., Wang J., Ge Q., Zhang H., Yang W., Sun X., Ma T. (2021). Shelf life of non-industrial fresh mango juice: Microbial safety, nutritional and sensory characteristics. Food Biosci..

[13] Georgiadou E.C., Antoniou C., Majak I., Goulas V., Filippou P., Smolińska B., Leszczyńska J., Fotopoulos V. (2021). Tissue-specific elucidation of lycopene metabolism in commercial tomato fruit cultivars during ripening. Sci. Hortic..

[14] Deng X., Mei X., Xu D., Cai Y. (2015). Niacin and Zeaxanthin Contents in Different Maize Kernels. Food Sci..

[15] Ma T., Wang J., Yang Y., Wang L., Yang W., Wang H., Lan T., Zhang Q., Sun X. (2020). Ultrasound-combined sterilization technology: An effective sterilization technique ensuring the microbial safety of grape juice and significantly improving its quality. Foods.

[16] Ismail B.B., Liu D., Pu Y., He Q., Guo M. (2021). High-intensity ultrasound processing of baobab fruit pulp: Effect on quality, bioactive compounds, and inhibitory potential on the activity of α-amylase and α-glucosidase. Food Chem..

[17] Lan T., Gao C., Yuan Q., Wang J., Zhang H., Sun X., Lei Y., Ma T. (2021). Analysis of the Aroma Chemical Composition of Commonly Planted Kiwifruit Cultivars in China. Foods.

[18] Nakamura H., Uchida S., Sugiura T., Namiki N. (2015). The prediction of the palatability of orally disintegrating tablets by an electronic gustatory system. Int. J. Pharm..

[19] Sun F., Zhu L., Wang X., Cheng J., Cui B., Liu J., Tan F., Fu M. (2020). Sucrose transportation control mediates the fresh-keeping effects of burdock fructooligosaccharide in ‘Crimson Seedless’ grapes. Food Chem..

[20] Machado I., Bergmann G., Pistón M. (2016). A simple and fast ultrasound-assisted extraction procedure for Fe and Zn determination in milk-based infant formulas using flame atomic absorption spectrometry (FAAS). Food Chem..

[21] Lemmens L., Vleeschouwer K., Moelants K., Colle I., Loey A., Hendrickx M. (2010). β-Carotene isomerization kinetics during thermal treatments of carrot puree. J. Agric. Food Chem..

[22] Lund M.N. (2021). Reactions of plant polyphenols in foods: Impact of molecular structure. Trends Food Sci. Technol..

[23] Knockaert G., Pulissery S.K., Lemmens L., Buggenhout S.V., Hendrickx M., Loey A.V. (2013). Isomerisation of carrot β-carotene in presence of oil during thermal and combined thermal/high pressure processing. Food Chem..

[24] Mapelli-Brahm P., Barba F.J., Remize F., Garcia C., Fessard A., Khaneghah A.M., Sant’Ana A.S., Lorenzo J.M., Montesano D., Meléndez-Martínez A.J. (2020). The impact of fermentation processes on the production, retention and bioavailability of carotenoids: An overview. Trends Food Sci. Technol..

[25] Ndolo V.U., Beta T. (2013). Distribution of carotenoids in endosperm, germ, and aleurone fractions of cereal grain kernels. Food Chem..

[26] Li X., Uddin M.R., Park W.T., Kim Y.B., Seo J.M., Kim S., Nou I., Lee J., Kim H.R., Park S.U. (2014). Accumulation of anthocyanin and related genes expression during the development of cabbage seedlings. Process Biochem..

[27] Ma T., Lan T., Ju Y., Cheng G., Que Z., Geng T., Fang Y., Sun X. (2019). Comparison of the nutritional properties and biological activities of kiwifruit (*Actinidia*) and their different forms of products: Towards making kiwifruit more nutritious and functional. Food Funct..

[28] Bai F., Wang Y., Zhang S., Wang Y., Zhang J., Cao J., Sun L. (2020). Caffeoyl substitution changes the inhibition mode of tartaric acid against α-amylase: Analysis of the enzyme inhibition by four caffeic and tartaric acid derivates. LWT Food Sci. Technol..

[29] Wang Y., Li S., Bai F., Cao J., Sun L. (2021). The Physical Adsorption of Gelatinized Starch with Tannic Acid Decreases the Inhibitory Activity of the Polyphenol against α-Amylase. Foods.

[30] Sun L., Warren F.J., Netzel G., Gidley M.J. (2016). 3 or 3′-Galloyl substitution plays an important role in association of catechins and theaflavins with porcine pancreatic α-amylase: The kinetics of inhibition of α-amylase by tea polyphenols. J. Funct. Foods.

[31] Gao Y., Liu Y., Kan C., Chen M., Chen J. (2019). Changes of peel color and fruit quality in navel orange fruits under different storage methods. Sci. Hortic..

[32] Xu J., Liu K., Zhang C. (2021). Electronic nose for volatile organic compounds analysis in rice aging. Trends Food Sci. Technol..

[33] Buratti S., Malegori C., Benedetti S., Oliveri P., Giovanelli G. (2018). E-nose, e-tongue and e-eye for edible olive oil characterization and shelf life assessment: A powerful data fusion approach. Talanta.

