# Supplementary material for: Research on the Consumption Trend, Nutritional Value, Biological Activity Evaluation, and Sensory Properties of Mini Fruits and Vegetables
Source: Foods. 2021 Dec 2;10(12):2966. doi: 10.3390/foods10122966 (PMC8700999; doi:10.3390/foods10122966)
Supplement: Supplementary file 1 [file foods-10-02966-s001.zip › foods-1457672-Supplementary.pdf]

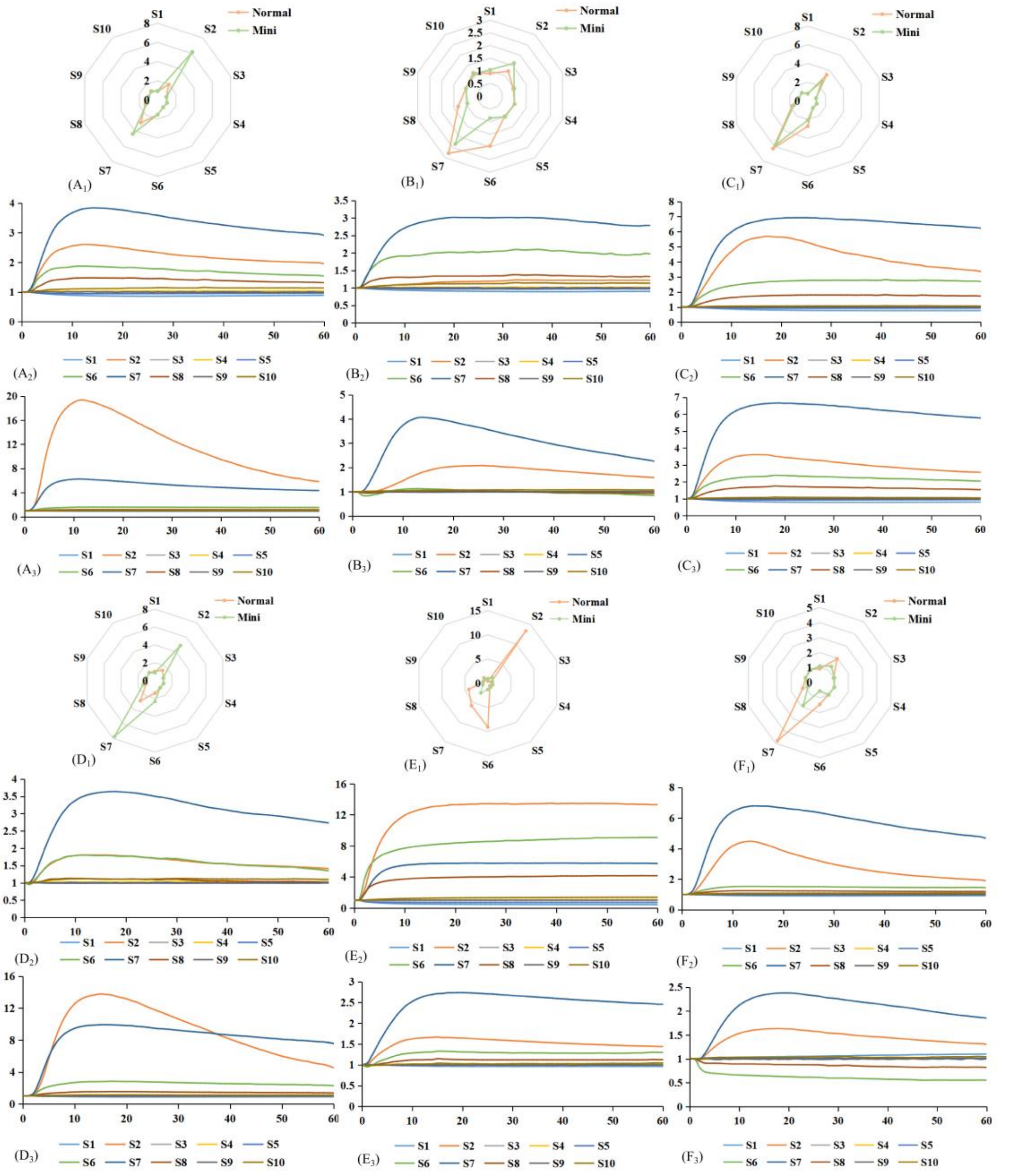

Figure S1. E-nose assay of MFV and NFV. (A_1-F_1) Radar charts of E-nose response data; (A_2-F_2) Sensor responses recorded using ten sensors of the E-nose for NFV; (A_3-F_3) Sensor responses recorded using ten sensors of the E-nose for MFV. (A. Watermelon; B. Pineapple; C. Pumpkin; D. Cabbage; E. Maize; F. Carrot.)

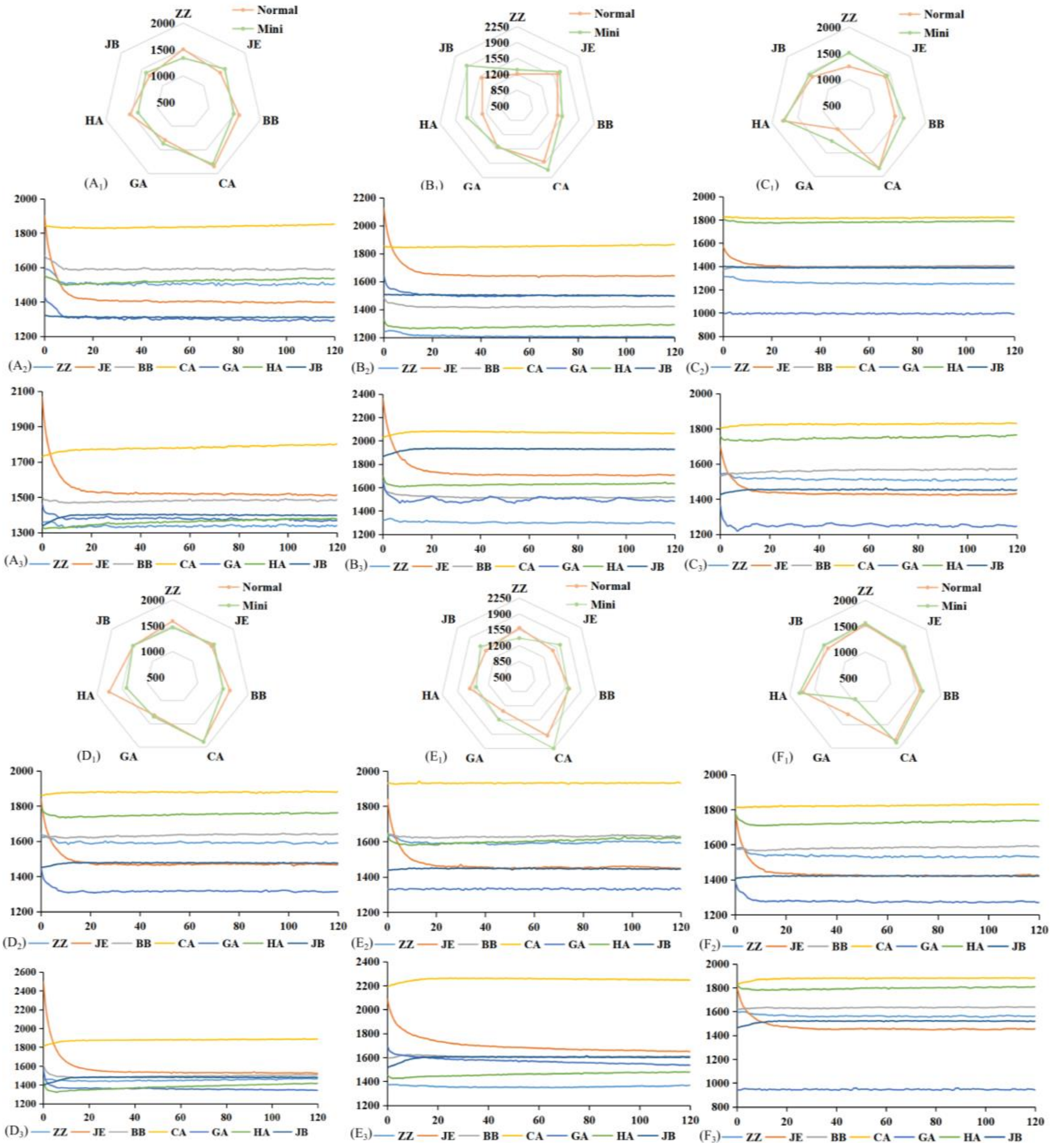

Figure S2. E-tongue assay of MFV and NFV. (A_1-F_1) Radar charts of E-tongue response data; (A_2-F_2) Sensor responses recorded using ten sensors of the E-tongue for NFV; (A_3-F_3) Sensor responses recorded using ten sensors of the E-tongue for MFV. (A. Watermelon; B. Pineapple; C. Pumpkin; D. Cabbage; E. Maize; F. Carrot.)

Table S1. Detection thresholds of each sensor of electronic tongue.

Basic taste	Sensor threshold (mol/L)						
	ZZ	JE	BB	CA	GA	HA	JB
Sour	10^{-7}	10^{-7}	10^{-7}	10^{-7}	10^{-7}	10^{-7}	10^{-7}
Sweet	10^{-7}	10^{-4}	10^{-7}	10^{-7}	10^{-4}	10^{-4}	10^{-4}
Salty	10^{-6}	10^{-5}	10^{-6}	10^{-6}	10^{-4}	10^{-4}	10^{-5}
Bitter	10^{-5}	10^{-4}	10^{-4}	10^{-5}	10^{-4}	10^{-4}	10^{-4}
Fresh	10^{-5}	10^{-4}	10^{-4}	10^{-5}	10^{-4}	10^{-4}	10^{-4}
